# Use of NOTES for endoscopic resection of a subphrenic mass initially diagnosed erroneously as originating from the muscularis propria

**DOI:** 10.1055/a-2410-3190

**Published:** 2024-09-25

**Authors:** Yanli Yu, Ye Wang, Wenxiao Zhao, Jihui Li, Ruobing Jiang, Mei Chen, Xiujing Zhang

**Affiliations:** 1 Department of Gastroenterology, North China University of Science and Technology Affiliated Hospital, Tangshan, China; 2 Department of Gastroenterology, Tianjin First Center Hospital, Tianjin, China

A 56-year-old woman presented with a protruding lesion located at the large curvature of the gastric fundal junction ( Fig. 1 ). Endoscopic ultrasonography showed a hypoechoic tumor with a clear boundary and an endoluminal growth pattern, and originating from the muscularis propria ( Fig. 2 ). Computed tomography images showed multiple, high-density, round lesions but no evidence of a lesion in the gastric wall ( Fig. 3 ).

**Fig. 1:**
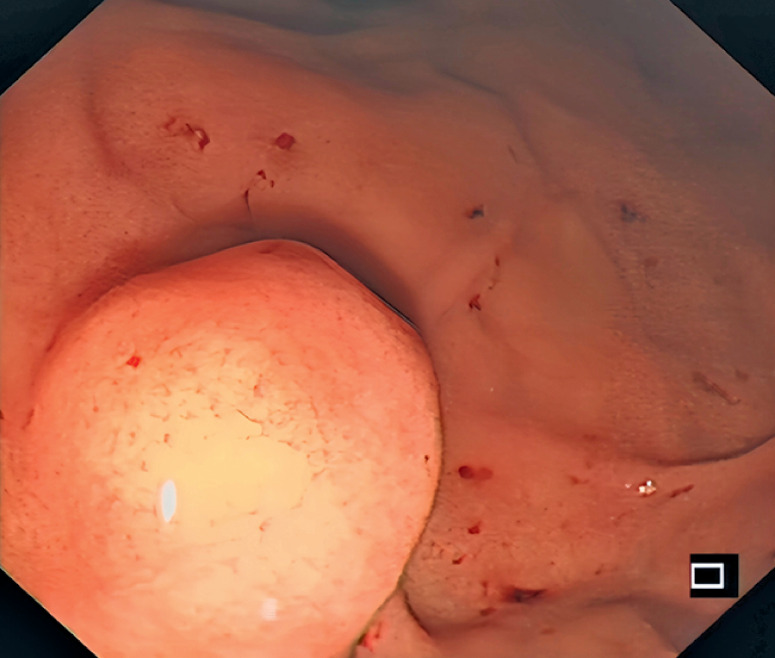
Endoscopic image showing a protruding lesion located at the large curvature of the gastric fundal junction.

**Fig. 2:**
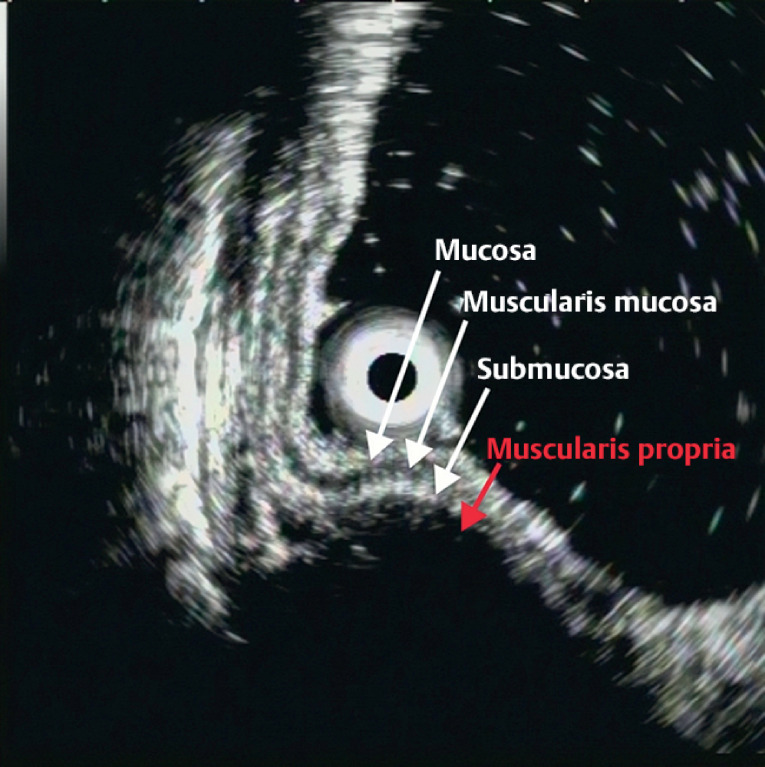
Endoscopic ultrasonography showed a hypoechoic tumor with a clear boundary and an endoluminal growth pattern, which originated from the muscularis propria.

**Fig. 3:**
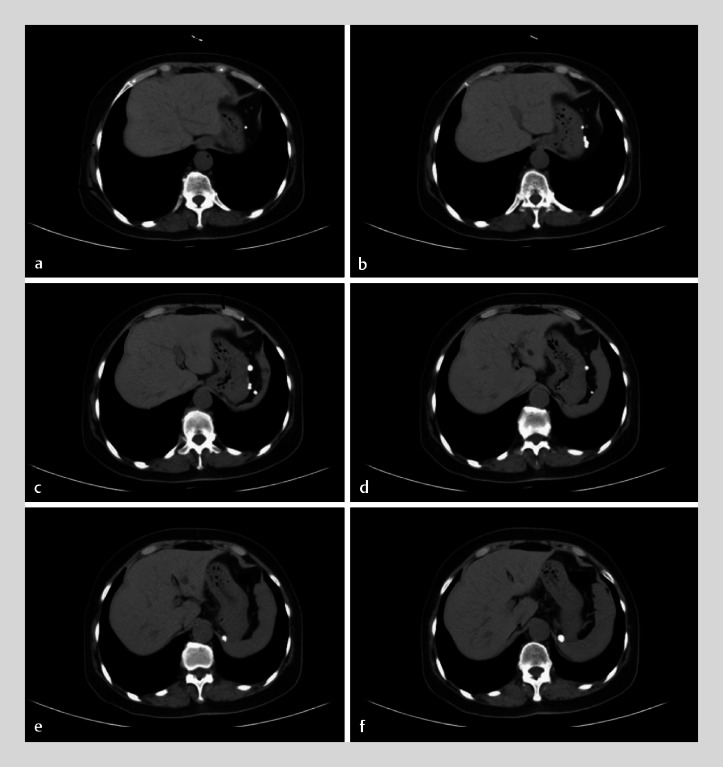
Computed tomography image series showing multiple, high-density, round lesions but no evident lesion on the gastric wall.

To achieve a deeper submucosal resection, endoscopic submucosal excavation (ESE) was performed. During the operation, the lesion was not found until the submucosal tissues, muscularis propria, and the serosal layer had been carefully dissected. We then switched to natural orifice transluminal endoscopic surgery (NOTES). When the endoscope passed through the ESE-produced perforation into the abdominal cavity, the extraluminal lesion was found. Within the peritoneal cavity, the lesion originated from the diaphragm and was surrounded by fibrous connective tissue ( Fig. 4 ). We resected the abdominal lesion with an insulation-tipped knife and a DualKnife (Olympus, Tokyo, Japan). Complete resection of the tumor was achieved. Adequate hemostasis was performed before closing the incision with clips ( Video 1 ).

**Fig. 4:**
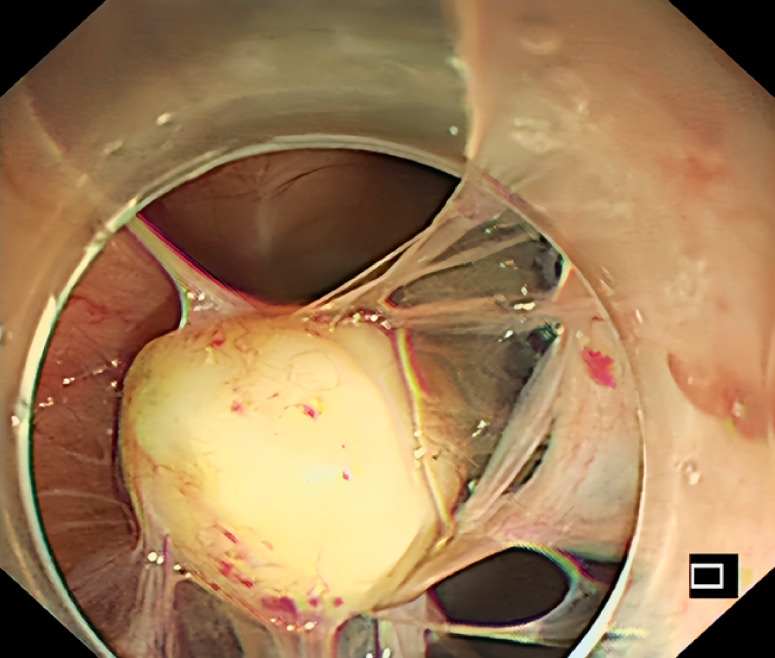
The lesion originated from the diaphragm and was surrounded by fibrous connective tissue.

Endoscopic resection of a subphrenic mass via natural orifice transluminal endoscopic surgery.Video 1

The histopathological analysis revealed collagen tissue arranged in a circular layer with calcification in the center, suggesting fiber calcifications ( Fig. 5 ). After surgery, no surgery-related complications were observed. Follow-up after 3 months and 7 months showed that the patient had no postoperative adverse events.

**Fig. 5:**
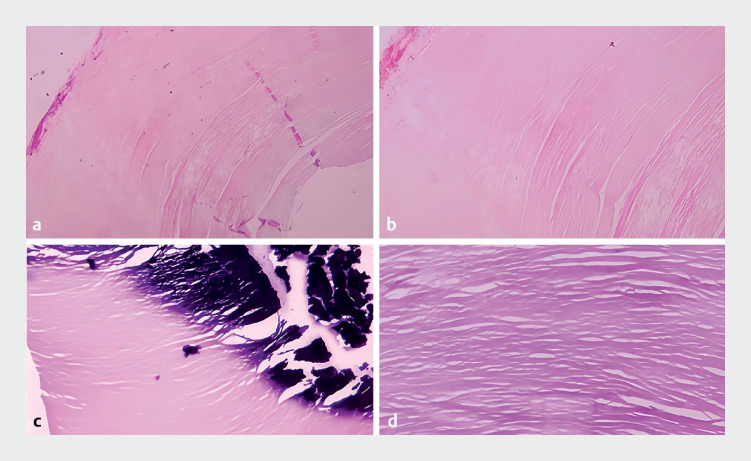
Histopathological findings (hematoxylin and eosin staining) suggested fiber calcifications. **a, b** Collagen tissue was arranged in a circular layer (×25 [ **a** ], ×50 [ **b** ]). **c, d** Calcification in the center (×50 [ **c** ], ×100 [ **d** ]).

In this case, we planned to perform ESE, but there was no tumor evident in the gastric wall until excavation reached full thickness. As some previous studies have reported the accuracy of EUS in determining the layer of tumor origin to be 75%–95% 1 , we also conducted CT, which together with gastroscopy suggested that the lesion may not be in the gastric wall. The use of transgastric NOTES not only clarified the diagnosis but also saved the patient from undergoing a second surgical operation 2 , which would have had implications for resourcing in the clinic.

In summary, endoscopic resection of a subphrenic mass via transgastric NOTES is novel and effective. Submucosal protruding gastric lesions originating from the muscularis propria layer on EUS should alert the endoscopist to the possibility of extragastric lesions closely connected to the gastric wall.

Endoscopy_UCTN_Code_CCL_1AF_2AG

## Notes

Endoscopy E-Videos https://eref.thieme.de/e-videos
*E-Videos* is an open access online section of the journal *Endoscopy* , reporting on interesting cases and new techniques in gastroenterological endoscopy. All papers include a high-quality video and are published with a Creative Commons CC-BY license. Endoscopy E-Videos qualify for HINARI discounts and waivers and eligibility is automatically checked during the submission process. We grant 100% waivers to articles whose corresponding authors are based in Group A countries and 50% waivers to those who are based in Group B countries as classified by Research4Life (see: https://www.research4life.org/access/eligibility/ ). This section has its own submission website at https://mc.manuscriptcentral.com/e-videos .
